# Tread Lightly Interpreting Polygenic Tests of Selection

**DOI:** 10.1534/genetics.118.300786

**Published:** 2018-03-29

**Authors:** John Novembre, Nicholas H. Barton

**Affiliations:** * Department of Human Genetics, Department of Ecology and Evolution, University of Chicago, Illinois 60637; † Institute of Science and Technology, A-3400 Klosterneuberg, Austria

**Keywords:** complex trait evolution, directional selection, polygenic

In this issue of GENETICS, a new method for detecting natural selection on polygenic traits is developed and applied to several human examples (Racimo et al. 2018). By definition, many loci contribute to variation in polygenic traits, and a challenge for evolutionary geneticists has been that these traits can evolve by small, nearly undetectable shifts in allele frequencies across each of many, typically unknown, loci. Recently, a helpful remedy has arisen. Genome-wide association studies (GWAS) have been illuminating sets of loci that can be interrogated jointly for changes in allele frequencies. By aggregating small signals of change across many such loci, directional natural selection is now in principle detectable using genetic data, even for highly polygenic traits. This is an exciting arena of progress – with these methods, tests can be made for selection associated with traits, and we can now study selection in what may be its most prevalent mode. The continuing fast pace of GWAS publications suggest there will be many more polygenic tests of selection in the near future, as every new GWAS is an opportunity for an accompanying test of polygenic selection. However, it is important to be aware of complications that arise in interpretation, especially given that these studies may easily be misinterpreted both in and outside the evolutionary genetics community. Here, we provide context for understanding polygenic tests and urge caution regarding how these results are interpreted and reported upon more broadly.

The foundations of polygenic tests of selection trace back to the very dawn of genetics, a century ago. An early challenge was to reconcile the inheritance of continuously distributed traits like height with the discretely inherited particulate genes of Mendelian inheritance. In a monumental advance in the history of genetics, R.A. Fisher reconciled this conflict by proposing that many genetic loci contribute to the variation of such traits – a polygenic model of inheritance – and by showing how to analyze their collective effect. In modern parlance, these traits are referred to as “quantitative,” “polygenic,” or “complex,” and each of the contributing loci are known as a quantitative trait locus (QTL). GWAS are revolutionary in allowing modern practitioners to uncover a fraction of the QTL that Fisher posited underlie most trait variation and to estimate effect sizes of each variant on a trait. Importantly for the discussion here, the polygenic model has evolutionary implications – most notably, the mean phenotypic value of a trait in a population can shift substantially and quickly via very subtle shifts in frequencies at the many QTL that underlie a trait. To see a signature of selection, modern polygenic tests use the QTL discovered by GWAS and detect shifts in their frequencies across time or across populations. The key advance has been to realize that while the impact of selection on any particular QTL cannot be reliably detected, the collective impact across many QTL can be.

The first polygenic selection study to use human GWAS results was based on a commonly studied trait, human height. Turchin *et al.* (2012) found a difference between northern and southern Europeans in the genetic component of height that appears to have been driven by differential selection across populations. A subsequent foundational paper by Berg and Coop (2014) gave a more formal analysis, by establishing a null model for how polygenic scores should vary across populations. This approach generalized and modernized a traditional pre-GWAS approach [comparison of Q_st_ *vs.* F_st_, Whitlock and Guillaume 2009] and has itself been extended to consider multiple traits (Berg *et al.* 2017). These initial approaches focused on comparison across populations, but comparisons can also be made across time within single populations using either ancient DNA (Mathieson *et al.* 2015) or using haplotypic signatures of allele frequency change (Field *et al.* 2016). The new study published in this issue by Racimo *et al.* provides a novel methodology for comparing across populations by using a population tree with migration edges (an “admixture graph”). The new method allows one to isolate which branches in an admixture graph have experienced polygenic selection. In some sense then, Racimo *et al.* extend Berg and Coop’s (2014) approach to allow more detailed resolution of when and where directional selection took place.

While these methodological contributions are important, the Racimo *et al.* study requires delicate handling. This is true for all polygenic selection studies, but all the more so here because of the comparison across populations for the complex behavioral trait of “educational attainment” (*i.e.*, years of schooling). Genetic contributions to educational attainment have been of keen interest to social scientists and economists who have an eye on implications for policy (e.g. Conley and Fletcher 2017). The Racimo *et al.* study is not unique in analyzing a sensitive trait with social implications – a recent study analyzes polygenic selection on 25 traits related to brain structure, neuropsychiatric characteristics, and personality traits (Beiter *et al.* 2017); more such studies should be expected. The recent publication of a GWAS of delay discounting (Sanchez-Roige *et al.* 2018) suggests that a polygenic selection test will soon be carried out too on this complex trait (delay discounting is the preference of an individual for an immediate reward rather than a larger reward after a delay). Such studies are particularly sensitive when they are based on between-population comparisons, as they draw attention to potential between-population differences in humans and suggest that these differences are not only detectable but also driven by selection that differs across populations. Given the potential for these results to affect social scientists and economists who may advise on policy making (e.g. Conley and Fletcher 2017), a clear understanding of the pitfalls of interpretation is especially necessary.

Here we want to emphasize that caution is needed as these results are discussed or considered in an applied context – there remain substantial challenges in interpreting results at the forefront of this innovative field. There are two main categories of complications. The first are the technical challenges that may give a false sign of directional selection. The second category are the complications of interpretation. We tackle each in turn.

A first technical concern is the potential for population stratification to affect the original GWAS study. The stratification problem is that a putative QTL from a GWAS study may just be a proxy for environmental variables or background genetic effects that have not been properly included in the statistical model. Suppose, for example, that environmental factors affecting a particular trait vary east to west across a study area. As a result, in a GWAS that is not carefully controlled for stratification, any variant that has differentiated in frequency along an east–west axis will have an overestimated effect size. In turn, a polygenic score would show elevated differentiation across populations at the QTL relative to a genome-wide background, even if selection has been absent. Any selection that differentiates populations along an east–west axis would produce similar spurious signals, even if it had nothing to do with the trait of interest. Correcting GWAS studies to avoid stratification has become standard using PCA and mixed model-based approaches (Price *et al.* 2010), but the concern of residual stratification still persists, and for this reason, some studies of polygenic evolution have relied on family-based estimates of effect sizes (Turchin *et al.* 2012; Field *et al.* 2016). The effect size estimates derived from within families are less susceptible to varying environmental factors but fewer within-family studies have been carried out – so this can be difficult to apply for most traits. LD-score regression (Bulik-Sullivan *et al.* 2015a) is an approach that allows one to assess whether residual stratification may be affecting a GWAS, and such approaches can also be useful for verifying polygenic signatures of selection (Field *et al.* 2016). As a practical matter, polygenic signatures that lack family-based estimates of effect size should be taken as provisional, given the concerns about residual confounding.

A second technical challenge is that of transferring effect sizes across space and time. Any GWAS is based on a particular study population sampled in the present-day. GWAS have been disproportionately carried out in European populations; a 2016 survey found that 81% of GWAS studies were from European ancestry populations (Bustamante *et al.* 2011; Popejoy and Fullerton 2016). The effects of this bias are unclear. GWAS loci are typically “tag SNPs” that indirectly reflect (“tag”) the effects of a nearby true causal QTL. The link between causal QTL and their tag SNPs may differ across space and time in ways that can bias polygenic scores. Further, there is the concern that the phenotypic effect of alleles at the QTL may differ by genetic or environmental background (due to epistasis and gene-by-environment interactions, G×G and G×E, respectively, in genetics shorthand). In practice, some analyses support the general transferability of GWAS across populations (Marigorta and Navarro 2013), while others suggest that problems arise in computing polygenic scores across populations (Martin *et al.* 2017). Given these uncertainties, unless the GWAS for a trait has been carried out in populations that are generally representative of the same studies used in the polygenic evolution study, one should be cautious in interpreting results. Current polygenic tests are narrowly testing whether the trait-associated SNPs found in a *particular* GWAS study population are evolving by drift across a possibly broader set of populations; the relevance for understanding how the *complete* genetic basis of a trait evolves will be unclear until more is learned about how the sharing and tagging of genetic effects can be modeled over time and space.

A third challenge is the ascertainment bias inherent in GWAS. SNPs identified by GWAS for traits of interest may be associated with regions that show “signatures” of selection simply because detection depends on allele frequencies, or less trivially, because SNPs that affect the trait also tend to affect fitness, for reasons that have nothing to do with the trait. For example, consider a disease trait studied in a European population – the ascertainment biases would make it such that discovered disease-risk alleles would have a systematically higher frequency inside Europe than outside, which could be mistaken for directional selection. In the studies discussed here, the signal is stronger: SNPs associated with increased trait values also tend to increase in frequency. Such directional associations give much stronger evidence for selection and show a genetic correlation between the trait and fitness. Nevertheless, as we discuss below, one still cannot say that the trait was *itself* selected (*i.e.*, that it *caused* increased fitness).

A final technical challenge specific to the Racimo *et al.* approach is that it depends on an accurate model of the populations and also, that there have been few directional selection events during the history of the trait. Notable violations of either of these assumptions may cause problems with the inference procedure, so careful evaluation of the validity of the admixture graph model should be undertaken when evaluating the results of any specific analysis. This is a pervasive problem in making inferences from genetic data: how can we know whether conclusions made assuming simplified models are valid, when there is an infinity of possible complications? The best that can be done is often to simulate a variety of plausible alternatives, but this can be computationally challenging.

Even if a signal of polygenic evolution is technically sound, there are still challenges to interpretation. First, many of the traits available for study by GWAS are awkward to conceptualize in an evolutionary framework. The majority of GWAS studies to date have been focused on traits of medical relevance or that are relatively easy to measure. As such, evolutionary geneticists are constrained to analyzing a somewhat arbitrary set of traits. No principled research program on understanding polygenic selection in human evolution would begin with traits such as self-reported unibrow or educational attainment. For educational attainment, the challenges are particularly acute; values observed today cannot be validly compared to those for humans living just 100 years ago when access to schooling was generally much more restricted, let alone to humans living 10,000 years ago. The caveat is also relevant when comparing across modern populations that differ greatly in the baseline schooling environment.

At the core there is a major challenge for interpretation in untangling the connection between measured traits (*e.g.*, height) and components of fitness (*e.g.*, increased success in survival, finding a mate, successful reproduction upon mating). For example, one may propose that height directly affects the probability of finding a mate; or alternatively, another in which height may be an indirect outcome of efficient metabolism, which has a direct impact on survival to adulthood. In either case, a polygenic signature of selection would be detected on height, but further multi-trait studies would be necessary to provide a precise interpretation as to whether height is directly or indirectly related to fitness. The key underlying problem is the existence of genetic covariance between traits (Lande and Arnold 1983). Traits can covary due to the presence of alleles that affect both traits (pleiotropy) or because of nonrandom association of alleles across loci (linkage disequilibrium). The potential for widespread pleiotropy due to the architecture of gene regulatory networks and gene action has been emphasized as part of an “omnigenic” model for complex traits (Boyle *et al.* 2017), and builds upon mounting empirical evidence of genetic covariance in human genetics (*e.g.*, Bulik-Sullivan *et al.* 2015b; Pickrell *et al.* 2015; Visscher and Yang 2016); the importance of pleiotropy has long been appreciated in quantitative genetics (*e.g.*, Barton 1990). New multivariate approaches to address polygenic traits are emerging (Berg *et al.* 2017) and new biobank resources are making analysis with direct measurements of fitness more feasible (Sanjak *et al.* 2018), but they still are complicated by the fundamental problem of missing, unmeasured traits (Lande and Arnold 1983). The challenges of trait covariance are complicated, even for the interpretation for some of the strongest single-locus signatures of selection (*e.g.*, EDAR; Kamberov *et al.* 2013). For these reasons, authors of polygenic studies, including Racimo *et al.*, are careful to say they have detected selection on “variants associated with trait X” and not “selection on trait X.” To truly say a trait is selected, *i.e.*, that it causes a difference in fitness, requires more direct evidence and extended study than allele frequency shifts alone can provide.

A second complication of interpretation regards comparisons across populations or time periods. Even when estimated correctly, relative values of a polygenic mean do not necessarily reflect relative values of the mean phenotypes that will be observed in populations. For instance, the polygenic mean for population A may be larger than B, but compensating environmental factors may make it such that population B has an equal or larger trait mean than in population A. A fascinating example of the potential disconnect between polygenic means and phenotypes can be seen in wild Soay sheep on the island of St. Kilda. Based on observations over 20 years, directional selection has been acting to increase the mean body size, yet body sizes have actually been decreasing, likely because of compensating effects in which recent changes in climatic conditions have led to slower growth rates and in turn lower average body sizes (Ozgul *et al.* 2009). This problem may not be idiosyncratic – many studies in natural populations have seen a disconnect between measured fitness differentials and observed phenotypic outcomes (Kruuk *et al.* 2008). For example, Robinson *et al.* (2015) show that in European humans, genetic differentiation among populations in body-mass index appears to be masked by environmental factors and that observed differences in average height are only partially explained by differentiation at current known GWAS loci. Another aspect of relative comparisons that is important to consider is that even when polygenic means differ, the variance of the distributions within populations may be so wide that there can be little predictive value in knowing which population an individual comes from.

Overall, the numerous complications described here, both technical and interpretative, are why this exciting field is still in its infancy. Progress is being made but most findings are wrapped in numerous caveats. For this reason, we caution that great care should be taken in communicating results of these studies to general audiences. Journalists producing simple headlines and/or taking results out of context have the potential to misconstrue the complexity and levels of uncertainty in an arena where simple misinterpretations come easily. Generally, authors of such studies, including Racimo *et al.*, are cautious, and this degree of caution must not be lost in translation. This is particularly sensitive as polygenic selection studies are analyzing complex social and behavioral traits, and as social scientists look more keenly upon these studies as a source of inspiration for policy; these efforts may be premature, to say the least. We are in a time of extraordinary discoveries, but we must remember that even as we gain traction with new computational tools and expanded genomic studies, we still have a long way to go to make precise, fully supported statements about the nature of selection on complex traits in humans.

Box 1 **Using GWAS to detect selection on traits**

The deviations of a trait, z, can be modeled as the sum of effects of a large number of SNPs: $z=\overset{}{\underset{i}{\sum }}\beta_{i}X_{i}{}_{}+\varepsilon$, where $X_{i}=0,1,2$ is an individual’s dosage of a reference allele at the ith SNP, $\beta_{i}$ is the effect of the SNP, and ε is a random “environmental” effect; effects of specific factors such as age, location, or sex can be represented by additional terms. The $\beta_{i}$ are estimated simply by regression of the trait on the genotype; call these estimates ${\hat{\beta }}_{i}$. Then, the genetic component of the trait value of a new individual with genotype $Y_{i}$, a “polygenic score”, can be estimated as $\hat{z}=\overset{}{\underset{i}{\sum }}{\hat{\beta }}_{i}Y_{i}{}_{}$. A difference between the mean $\hat{z}$ values for different sets of individuals can suggest selection on the trait if the difference is larger than expected by neutral evolution (i.e. genetic drift). Such selection may act through small shifts in allele frequency, which can nevertheless accumulate over very many loci to cause a large change in the trait mean.

Shifts in mean polygenic score may have many causes, in addition to selection on the observed trait:

- Selection on other traits.
- Selection may act on a trait that is genetically correlated with the observed trait, for instance through pleiotropic side-effects of the underlying alleles.
- Stratification: If the GWAS uses a sample that is a mixture of individuals from different places or different social groups, and the trait varies among these groups, then SNPs that are differentiated between groups can be spuriously associated with the trait.
- Varying effects: Changes in effects, β, between populations will tend to reduce the signal.
- Ascertainment bias: Common SNPs are more likely to be ascertained as significant in GWAS, and this may bias allele frequency differences that are the basis for inferring selection. However, a systematic directional change in frequency of alleles associated with increases in the trait gives a more robust signal.
- Random drift gives the null model: Its effects can be simulated, but an appropriate model must be chosen, which takes account of both population structure and linkage.
